# Comparison of bilateral to unilateral total extra-peritoneal (TEP) inguinal hernia repair: a systematic review and meta-analysis

**DOI:** 10.1007/s10029-023-02785-0

**Published:** 2023-04-03

**Authors:** T. Hitman, A. S. R. Bartlett, A. Bowker, J. McLay

**Affiliations:** 1 School of Medicine, University of Auckland, Auckland, New Zealand; 2 Department of Surgery, University of Auckland, Grafton, Auckland New Zealand; 3 Department of General Surgery, Auckland City Hospital, Grafton, Auckland New Zealand; 4 Laparoscopy Auckland, Epsom, Auckland New Zealand; 5 Faculty of Science, Statistics, University of Auckland, Auckland, New Zealand

**Keywords:** TEP, Inguinal, Hernia repair, Laparoscopic surgery

## Abstract

**Purpose:**

Laparoscopic herniorrhaphy (LH) has become the treatment of choice in many centers for patients with inguinal hernia (IH). Our aim was to compare the morbidity outcomes of bilateral vs unilateral IH repair using the laparoscopic total extra-peritoneal (TEP) technique, to determine whether undertaking bilateral IH repair places patients at additional risk.

**Methods:**

Manuscripts published up to the end of 2021 on PubMed/MEDLINE, EMBASE, Cochrane Library, Scopus, and Web of Science were searched. Patients (> 16 years) undergoing a primary elective unilateral or bilateral TEP operation, using the standard 3-port laparoscopic technique, were identified. Quality of evidence was assessed using the GRADE criteria. Meta-analysis was conducted where possible. Where this was not possible, vote counting was conducted using effect direction plots.

**Results:**

Eight observational studies, with a total of 18,153 patients were included. Operative time was significantly longer for bilateral operations. There was no significant difference in conversion to open, post-operative seroma, urinary retention, haematoma, and length of hospital stay. There was an increased rate of hernia recurrence in patients undergoing bilateral IH repair.

**Conclusion:**

Although limited by the observational nature of the included studies, there is no conclusive evidence to suggest a differential burden of morbidity between unilateral and bilateral TEP IH repair. As all included papers are from observational studies only, evidence from all outcomes is at best very low quality. This manuscript thereby highlights a need for randomized controlled trials to be conducted in this area.

**Supplementary Information:**

The online version contains supplementary material available at 10.1007/s10029-023-02785-0.

## Introduction

Inguinal hernia repair is one of the most commonly performed surgical procedures worldwide [1, 2]. Increasingly patients are having their hernia repaired laparoscopically, either by a transabdominal preperitoneal (TAPP) or total extra-peritoneal (TEP) technique. One advantage shared by both laparoscopic techniques is that they allow for concurrent repair of the contralateral side without the need for further incisions.

The literature, including large population-based studies [3, 4], shows minimal differences between outcomes of bilateral and unilateral TEP operations. Current perceived disadvantages of bilateral TEP are attributed to increased intraoperative and postoperative complications [3, 4], and in some cases an elevated risk of urinary bladder injuries [4], higher reoperation rate [4], as well as short-term postoperative pain/discomfort [5]. However, there have been no systematic reviews conducted on the topic, nor has there been an individual journal article sufficiently conclusive to address the comparison of operation outcomes in patients undergoing unilateral and bilateral IH repair.

The aim of this study was to undertake a systematic review and meta-analysis to address whether bilateral TEP IH repair is associated with increased morbidity compared to unilateral repair.

## Methods

### Eligibility criteria

The population of interest were adult patients (> 16 years) undergoing an elective unilateral or bilateral TEP operation for a primary IH. Patients with history of previous lower abdominal surgery, uncorrected coagulopathy, as well as those unfit for general anaesthesia were excluded. This review exclusively focused on the standard 3-port TEP laparoscopic technique for unilateral and bilateral IH repair. Variations in TEP technique (eg. needlescopic, single incision) were excluded. The primary outcome of interest was patient morbidity, reported through intraoperative and postoperative complications. Secondary outcomes were length of hospital stay, operative time, recurrence, post-operative pain and time to return to work. This review limited its scope to quantitative papers (RCTs and prospective/retrospective observational studies). Non-English language papers and studies with less than 10 participants were excluded.

### Grouping of studies for synthesis

Meta-analysis was considered in all instances where two or more articles reported data on the same outcome. Where this threshold was not reached or where outcomes were heterogenous, an effect direction plot was conducted instead. In these plots, outcomes were grouped by their underlying similarity, eg. intraoperative bleeding and intraoperative injuries as contributors to the category: intraoperative complications.

### Information sources

All manuscripts published up until the end of September 2021 and indexed in the following databases were searched: PubMed/MEDLINE, EMBASE, Cochrane Library, Scopus, and Web of Science. No attempt was made to gather unpublished data. Databases were last searched on the following dates: PubMed-30/10/21, MEDLINE, EMBASE-01/11/21, Cochrane Library, Scopus, Web of Science-03/11/21.

### Search strategy

The search string used in PubMed/Medline was (Inguinal AND hernia AND (repair OR hernioplast*)) AND (TEP OR "-“totally extraperitoneal” OR “total extraperitoneal” OR “total extra-peritoneal”) AND ((Unilateral AND Bilateral) OR (“unilateral vs bilateral” OR contralateral* OR simultaneous* OR sequential*)) AND (Outcome* OR morbidity OR “quality of life” OR QoL OR recurrence OR ((postoperative OR intraoperative) AND complication*)) AND (“Hernia, Inguinal”[Mesh] OR “Herniorrhaphy”[Mesh]) AND “Laparoscopy”[Mesh] AND (“Treatment Outcome”[Mesh] OR “Intraoperative Complications”[Mesh] OR “Postoperative Complications”[Mesh] OR “Pain, Postoperative”[Mesh] OR “Recurrence”[Mesh] OR “Quality of Life”[Mesh]).

The search string was adapted to Embase and is available in the protocol [6]. Where an indexing function was not available (Scopus, Web of Science) only keywords were used. An attempt was made to gather further references by cross-checking all reference lists manually from articles.

### Selection process

Search results yielded from each search string were downloaded as separate bibliography files stored within Mendeley Reference Manager version 2.69.0 [7]. At minimum the following parameters were filled: author names, publication year, journal, DOI, URL link, and abstract. Deduplication of articles was conducted in Zotero version 5.0.96.3 [8].

Screening was conducted by one reviewer. Any uncertainties in article selection were adjudicated through discussion with two third parties.

### Data collection process

Data was extracted manually from included studies by one reviewer using Excel version 2108.

### Data items

All quantitative measures of intraoperative and postoperative morbidity, as well as features of the operation such as operative duration, assessments of postoperative pain and quality of life, and measures of patient convalescence reported by studies were extracted. All reported outcome parameters from the included pool of studies were tabulated. Definitions for outcomes which had syntheses conducted are presented in Supplementary Table 1.

Data were extracted regarding the general features of each study, including the study year, study authors, features of the study setting such as location and hospital, as well as information describing the study design. Further data was extracted for demographic and participant information, including the total number of participants, the number of participants present in each study arm, mean patient age, ratio of male to female sex, mean American Society of Anaesthesiologists (ASA) score, and follow-up duration.

The majority of studies did not supply information about ASA grading. It was assumed that this would not significantly affect the measures of postoperative morbidity reported.

### Study risk of bias and certainty assessment

The quality of evidence was judged both within and across studies in accordance with the GRADE criteria [9]. The five domains of the criteria include risk of bias, consistency, directness, precision and publication bias, aggregating to form one certainty score. Included studies were examined for their application of eligibility criteria, control of confounders, differential surveillance for outcomes and appropriateness of follow-up duration. An assessment of inconsistency was derived from the existence of a wide variation in point estimates, a lack of confidence interval (CI) overlap and tests of statistical heterogeneity. Indirectness was based on the relevance of each paper to the predefined PICO. A verdict of imprecision was reached based on whether or not the Optimal Information Size (OIS) was met, if the 95% CI overlapped no effect and whether or not the 95% CI excluded reasonable benefit or harm (boundaries set at a RR/ROM of 0.75 and 1.25, respectively). OIS was calculated for dichotomous outcomes and continuous outcomes [10] assuming an alpha of 0.05 and a beta of 0.20. Critical appraisal of the included studies was conducted by one reviewer.

### Synthesis methods

Quantitative synthesis was conducted with the assistance of a Statistical Consultant. The software package RStudio version 4.1.2 [11] was used for statistical analysis to produce a combined outcome. Meta-analysis was conducted following the methodology of Balduzzi et al. [12]. Both forest and funnel plots were constructed. Dichotomous outcomes were calculated in the form of Risk Ratio (RR) with 95% confidence intervals (CI). The Mantel–Haenszel method [13, 14] was used for pooling the studies. Both fixed and random effects models were run but the random effects model was considered the primary output. Studies with zero events in both the unilateral and bilateral group were excluded from the analysis in accordance with Cochrane guidelines [15]. Continuous outcomes were calculated in the form of Mean Difference (MD), as well as Ratio of Means (ROM) where justified, using 95% confidence intervals (CI). Inverse variance weighting was used for pooling the studies.

For studies Bochkarev et al. (2007) [16] and Kebabci et al. (2021) [17] the standard deviations for mean operative time were not available, only minimums and maximums, and so Eqs. 16 and 9 in Wan et al. (2014) [18] were used to estimate the missing standard deviations.

Where it was not possible to conduct a meta-analysis of effect estimates, vote counting was conducted using effect direction plots in accordance with the methodology described by Boon and Thomson (2020) [20]. A breakdown of contributing outcomes to each effect direction plot is provided in Supplementary Table 4 and Supplementary Table 5 for transparency. As formal assessments of heterogeneity are not possible for vote counting, this was restricted to consideration of possible clinical heterogeneity between included studies.

## Results

### Study selection

**Fig. 1:**
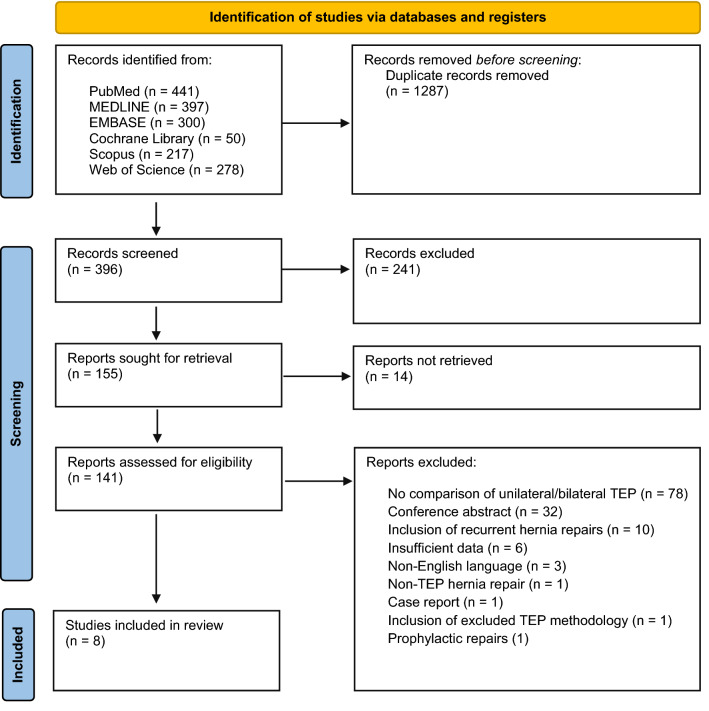
PRISMA flow diagram of the included studies

The results of the study selection process are represented in a PRISMA flow diagram (Fig. 1). The search strategy identified 1683 papers. Following deduplication, 396 papers remained and were subjected to the title and abstract screening criteria. Of this pool of papers, 155 papers showed, or appeared to show, a comparison between unilateral and bilateral TEP operations. Upon full-text examination, only 26 of these articles contained data comparing technique outcomes. A further 18 articles from this group were excluded due to contamination with recurrent hernia repairs, prophylactic repairs, or an excluded TEP methodology, leaving 8 articles [3, 4, 16, 17, 19, 21–23], with a total of 18,153 patients, included in the final analysis.

There were six studies [24–29] excluded for “insufficient data”. These were papers in which the inclusion criteria was technically met, however the comparison between unilateral and bilateral TEP outcomes were of secondary or incidental interest. These papers lent themselves to a high degree of indirectness as the data being presented was unique to or incompatible with the body of evidence that emerged in the final stages of selection. Consensus was reached among reviewers that these studies should be excluded on the basis of insufficient data.

The study conducted by Lal et al. [30] met the eligibility criteria, but included a sub-population of bilateral repairs which were conducted prophylactically for unilateral patients. As no hernia sac was present on the contralateral side, a true comparison for the outcomes of operative time, seroma formation, post-operative pain, and recurrence could not be made and this paper was therefore excluded.

Studies which included repairs of contralateral occult hernias [16, 17, 19, 23] discovered intraoperatively, via imaging, or clinically, were excluded from analysis regarding pain and convalescence. The literature has demonstrated that patients who have more pain in the preoperative period are also more likely to have more pain in the postoperative period [31]. Patients with symptomatic hernias would therefore be more likely to experience postoperative pain and worse convalescence than occult hernias, which impedes on the comparison of unilateral and bilateral operations.

One of the exclusion criteria was the presence of TEP combined with another operation. The population reported by Lau et al. (2003) [21] includes the presence of concurrent operations alongside TEP in a minor subset of the population (n = 6/206). These operations consist of two instances of a Jaboulay operation, a laparoscopic cholecystectomy, paraumbilical hernia repair, hemorrhoidectomy, and excision of an elbow mass. As these operations occurred in only a minor subset of their sample, consensus was reached among reviewers that this paper should be included.

### Study characteristics

The study characteristics and pre-operative patient data for the included studies (n = 8) are presented in Supplementary Table 2.

### Critical appraisal

The results of GRADE critical appraisal are displayed in the evidence and summary of findings table Supplementary Table 3. As all studies have an observational design, they are graded as low certainty by default. A high degree of directness to the specified PICO was observed throughout all studies. The risk of bias in the included studies was assessed. Eligibility criteria were well developed and applied throughout, selection bias was not detected, differential surveillance for outcomes was not apparent. Prognostic information (eg. ASA grade) was largely unavailable in studies and may have been unequally distributed between unilateral and bilateral study arms. The shortest mean follow-up duration of seven months was considered adequate. All studies were assessed as having a low or uncertain risk of bias. Where risk of bias was uncertain, this was determined to have a non-critical impact.

To assess publication bias, funnel plots were interpreted for each meta-analysis. However, in accordance with Cochrane guidelines [32], more than ten studies would be needed to contribute to the funnel plot for this to distinguish chance from real asymmetry. There were no other considerations of note, and none of the outcomes met the criteria for rating up a level.

### Operative time

**Fig. 2:**
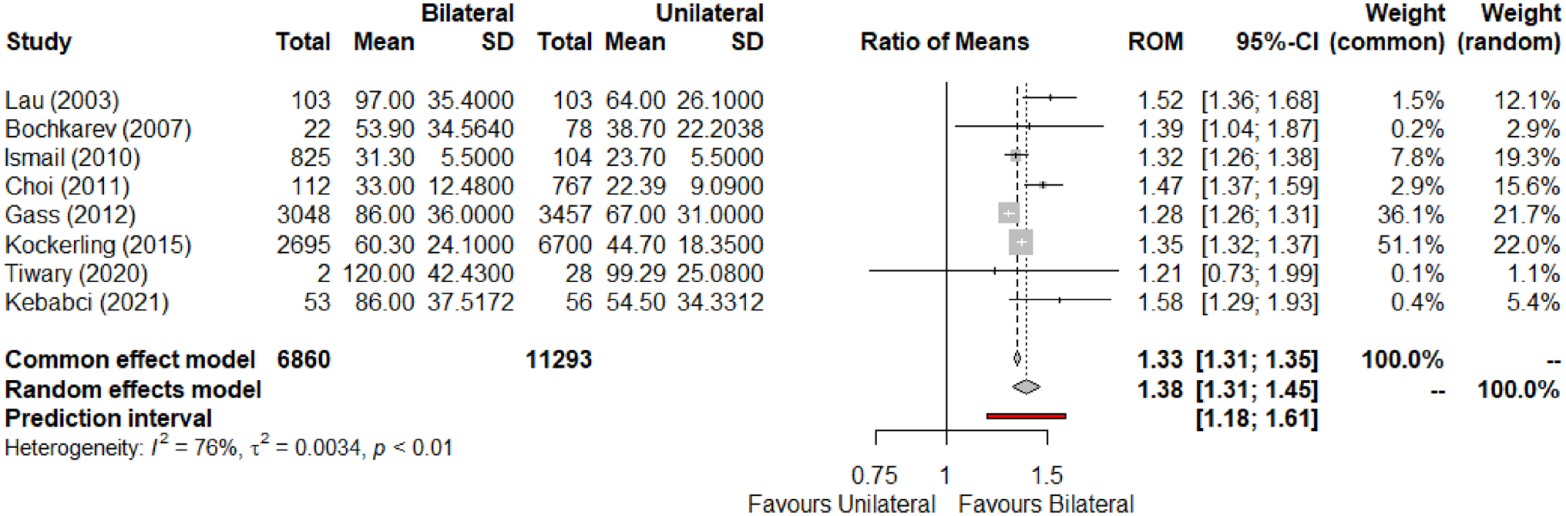
Forest plot for mean operative time

All included studies [3, 4, 16, 17, 19, 21–23] reported operative time (n = 18,153). The duration of bilateral IH repair was universally greater than that of unilateral repairs. Meta-analysis shows a mean difference of 17.97 min (95% CI, 11.08–24.87; Z = 5.11, P < 0.0001) between operations. In addition, a ratio of means of 1.38 could be attributed to this relationship (CI, 1.31–1.45; Z = 11.66, P < 0.0001) (Fig. 2). There was significant heterogeneity among the contributing papers (MD: I^2^ = 96%, τ^2^ = 74.09, p < 0.01; ROM: I^2^ = 76%, τ^2^ = 0.0034, p < 0.01). Hence, the GRADE domain of inconsistency was relevant in critical appraisal of this outcome. Small differences in point estimates of large papers may be influencing this statistic. It is also possible that variations in experience and surgical efficiency with the second component of bilateral operations may be contributing to clinical heterogeneity. Holistically, this element of inconsistency was not considered serious. Synthesis performed in the relative measure (ROM) was considered the primary output due to decreased heterogeneity and improved predictability when applied to a given unilateral repair duration. The certainty in this outcome was graded as low, due to being derived from observational studies.

### Conversion to open

The incidence of conversion to open was reported by two studies [3, 21] (n = 7,434). Meta-analysis shows the occurrence of conversion being similar in bilateral and unilateral IH repair (RR: 1.08; 95% CI, 0.67–1.75; Z = 0.33, P = 0.7384). Statistical heterogeneity between contributing studies was not apparent (I^2^ = 0%, τ^2^ = 0, P = 0.7241). The certainty of this outcome was downgraded from low to very low for imprecision, as the OIS was not met, nor was the 95% CI able to exclude important benefit and harm.

### Intraoperative complications

Other than conversion to open, no outcomes were held in common by the included studies. Hence, further meta-analyses were not conducted. Three studies [3, 4, 21] reported data on intraoperative complications. The effect direction plot (Table 1) showcases one of the three studies reporting increased intraoperative complications during bilateral procedures, one with unilateral procedures, and the remaining study reporting conflicting or unclear effects. This outcome was graded as being of low certainty, due to being derived exclusively from observational studies.

**Table 1:** Effect direction plot

Author	Study design	Intra-op complications	Post-op complications	Recurrence
Lau (2003)	CS	▲	▼	▼
Bochkarev (2007)	CS		◄►2	▼
Ismail (2010)	CS			▲
Choi (2011)	CS			▼
Gass (2012)	PBA	▼	▼2	
Köckerling (2015)	PBA	◄►5	◄►6	
Tiwary (2020)	CS		▼	
Kebabci (2021)	CS		▲2	▼

Study design, *CS* Cohort Study, *PBA* Population-Based Analysis. Effect direction upward arrow ▲ = favours unilateral, downward arrow ▼ = favours bilateral, sideways arrow ◄► = no change/conflicting findings. Subscripts indicate number of contributing outcomes. Intervention group sample size:▲ > 300, ▲ 50–300, ▲ < 50

### Seroma

**Fig. 3:**
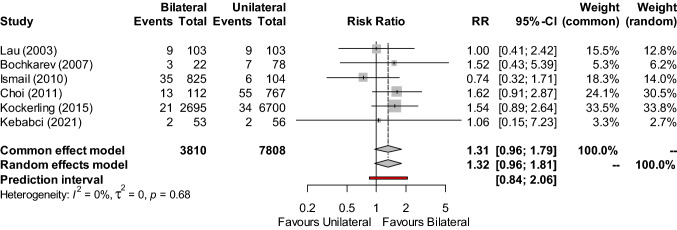
Forest plot of seroma occurrence

The incidence of seroma was synthesized from the data of six studies [4, 16, 17, 21–23] (n = 11,618) (Fig. 3), which showed similar occurrence following both unilateral and bilateral repairs (RR: 1.32; 95% CI, 0.96–1.81; Z = 1.72, P = 0.0860). This result was borderline in favour of increased seroma occurrence following bilateral repairs, however statistical significance was not reached. There was no statistical heterogeneity (I^2^ = 0%, τ^2^ = 0, P = 0.6809). The certainty of this outcome was rated down from low to very low for imprecision, due to its 95% CI being unable to exclude important harm.

### Urinary retention

**Fig. 4:**
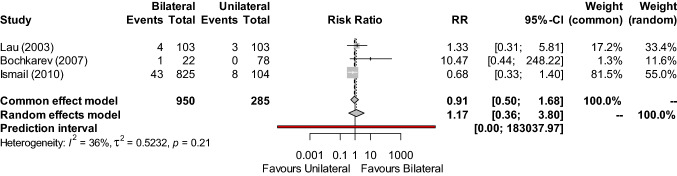
Forest plot of urinary retention occurrence

Urinary retention was reported by three studies [16, 21, 22] (n = 1,235) (Fig. 4), and showed similar occurrence following both unilateral and bilateral IH repair (RR: 1.17; 95% CI, 0.36–3.80; Z = 0.26, P = 0.7978). Measures of statistical heterogeneity were low (I^2^ = 36%, τ^2^ = 0.5232, P = 0.21). The certainty in this outcome was downgraded from low to very low for imprecision, as the calculated OIS was not met, and the 95% CI was unable to exclude both important benefit and harm. In addition there was a serious occurrence of inconsistency, due to the small study effect evident in the data of Bochkarev et al. (2007) [16].

### Haematoma

**Fig. 5:**
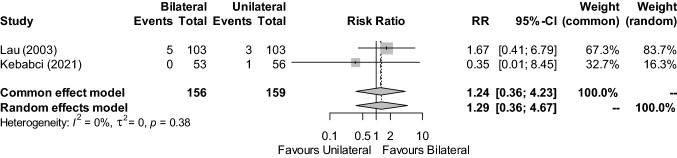
Forest plot of haematoma occurrence

Two studies reported the incidence of haematoma [17, 21] (n = 315) (Fig. 5). The risk of haematoma was similar in recipients of bilateral and unilateral repairs (RR: 1.29; 95% CI, 0.36–4.67; Z = 0.39, P = 0.6954). There was no statistical heterogeneity between contributing studies (I^2^ = 0%, τ^2^ = 0, P = 0.3805). The certainty in this outcome was downgraded from low to very low for imprecision, as the calculated OIS was not met and the 95% CI was unable to exclude both important benefit and harm.

### Miscellaneous postoperative complications

Further postoperative complications were reported by the included studies, but these were either unique or unable to be synthesized due to there being zero events in both study arms. Six studies [3, 4, 16, 17, 19, 21] reported data of miscellaneous postoperative complications. In the effect direction plot (Table 1), three studies reported increased postoperative complications following bilateral operations, one study saw increased postoperative complications following unilateral operations, and the remaining two studies had conflicting/unclear results. This outcome is graded as being of low certainty, as all contributing studies are observational in nature.

### Recurrence

While six of the included studies reported hernia recurrence, a meta-analysis could not be conducted for this outcome. Clinical heterogeneity is present in regard to significant variation in mean follow-up duration, ranging from seven to 38 months. In one study [17] follow-up duration was not reported. In a further study [19] zero events were reported in both study arms, preventing synthesis. Five studies [16, 17, 21–23] contributed to an effect direction plot for this outcome (Table 1). Four of the five studies saw an increased rate of recurrence following bilateral repair compared to unilateral repair, with the effect estimate of Ismail et al. (2010) [22] being skewed due to asymmetrical recruiting. The certainty in this outcome was rated down from low to very low due to inconsistency with significant variation in follow-up periods between included studies.

### Length of hospital stay

**Fig. 6:**
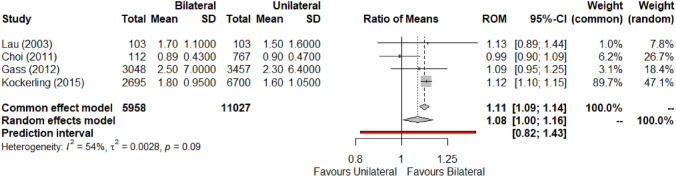
Forest plot for mean length of hospital stay

Four studies [3, 4, 22, 23] contributed to a meta-analysis for length of hospital stay (LOHS). In the conducted meta-analyses, there was no statistically significant increase in LOHS in the bilateral group compared to unilateral group (MD: 0.12; 95% CI, − 0.02–0.69; Z = 1.73, P = 0.0836; ROM: 1.08; 95% CI 1.00–1.16; Z = 2.07, P = 0.0382) (Fig. 6). In this synthesis the point estimates vary between studies, with approximately a 0.25-point gap between largest and smallest point estimates. Heterogeneity is significant (MD: I^2^ = 85%. τ^2^ = 0.0030; ROM: I^2^ = 54%. τ^2^ = 0.0028). Consequently, the GRADE domain of inconsistency is relevant to this outcome. Differing discharge protocols at the contributing hospitals may be influencing this heterogeneity. The majority of contributing studies treated TEP hernia repair as a day case. However, Gass et al. (2012) [3] had a mean length of stay above two days for both unilateral/bilateral procedures, which varies significantly from all other contributing papers. The outcome as ROM is favoured due to marginal improvement in heterogeneity. In addition, a relative measure may be more applicable in the clinical setting due to variations in discharge protocol with hernia repair. Inconsistency was disregarded in the certainty assessment for this outcome, which remains at low.

## Discussion

Laparoscopic IH repair using the TEP technique is a safe, well-established, and recommended surgical technique for inguinal hernia repair [33–36]. The outcomes of simultaneous TEP repair compared to sequential open hernia repair have historically been documented as being very similar [37–39], indicating that additional surgical trauma and operative time with bilateral repair does not significantly affect morbidity or recovery parameters. Contrary to this, laparoscopic procedures using the TAPP technique appear to show at best comparable [40], but more often worse outcomes following bilateral procedures [41, 42]. Jacob et al. (2015) [42], in their population-based analysis of 15,176 operations, reported a surgical postoperative complication rate of 4.9% for bilateral repairs compared to 3.9% for unilateral repairs, which can be attributed to a significantly higher rate of intestinal obstructions. This present meta-analysis has evaluated the outcomes of bilateral compared to unilateral hernia repair using the laparoscopic TEP technique.

Given the opportunity to operate bilaterally through the same incisions, several authors have discussed the merits of performing prophylactic bilateral TEP repairs in patients with clinically unilateral inguinal hernias [30, 43–47]. A rationale for this argument is based on the occurrence of contralateral hernia following unilateral repair, and the postulated benefits that may be gained for the patient and healthcare system through the administration of one anaesthesia, operation and recovery period.

Current interventions in place to address the possibility of metachronous contralateral hernia include intraoperative contralateral exploration, which is considered a significant advantage of laparoscopic hernia repair compared to open techniques. At present, contralateral exploration is advocated by many as routine practice [16, 48, 49]. However, even in patients with negative contralateral exploration a symptomatic metachronous hernia may still develop [47]. Zendejas et al. (2011) [43] report a 1.2% yearly risk of developing a contralateral hernia following negative exploration.

No clear consensus or recommendations have been established in regard to prophylactic repair, with some authors in opposition to this idea in accordance with their respective evidence [4, 45, 47]. Indiscriminate prophylactic repairs are largely cautioned against, however some authors argue for a utility in particular populations where benefit may outweigh theoretical risks, for instance in elderly populations where additional anaesthesia may be problematic [45], or in disadvantaged rural settings where healthcare may not be as available [22]. This meta-analysis may provide some guidance in this discussion.

This systematic review and meta-analysis has demonstrated that bilateral TEP repairs require a longer operative duration than unilateral TEP repairs. One valuable outcome of this meta-analysis is that a generalized estimate of a 38% increased operative duration can be attributed to this relationship. No significant differences between unilateral and bilateral repairs could be discovered for any morbidity parameters explored. However, it should be noted that the meta-analysis of seroma occurrence was of borderline statistical significance in favour of bilateral repairs (RR = 1.32, 95% CI 0.96–1.81, Z = 1.71, P = 0.0870). No significant difference could be seen in the meta-analysis for length of hospital stay, however this outcome was also of borderline statistical significance in favour of bilateral repairs (ROM: 1.08; 95% CI 1.00–1.16 Z = 2.07, P = 0.0382).

Where meta-analysis was not conducted, effect direction appears to show a mixed pattern in the studies reporting intraoperative complications during bilateral and unilateral repairs. A closer inspection of the contributing data shows significant discrepancy in population size and variation in assessed outcomes between studies. Population-based studies suggest elevated rates of intraoperative injuries and unspecified complications during bilateral repairs, but also note an increased rate of bleeding with unilateral repairs. Postoperative complications that were unable to be synthesized via meta-analysis holistically show mixed or worse outcomes following bilateral repairs. Further studies reporting on the same intraoperative and postoperative outcomes are required to reach a convincing level of evidence in this regard.

Recurrences appeared to occur more frequently following bilateral repairs compared to unilateral repairs. A greater proportion of recurrences were seen in the bilateral group for all studies reporting data on this metric, sparing Tiwary et al. (2020) [32] in which no events were observed for either group.

A significant limitation of this study can be attributed to the inclusion of only observational studies. Applicable data from randomized controlled trials is currently non-existent in this field of research, which severely limits the strength of conclusions and recommendations that can be made. Due to being derived solely from observational studies, the quality of evidence of all outcomes synthesized in this review is at best very low quality. The comparison of unilateral and bilateral outcomes following laparoscopic repair has important implications for patient care. Many centres are routinely carrying out laparoscopic IH repair, despite the scarcity of literature assessing the comparison of bilateral and unilateral repair outcomes. This review highlights that further research in this area through randomized controlled trials is justified to improve the level of evidence available.

Statistical limitations of this review can be seen through the degree of imprecision in the summary effect estimates of seroma, haematoma and urinary retention outcomes. Limitations are evident in the synthesis method of vote counting using effect direction plots. Contributors to outcomes are not weighted and may give equal precedence to minor findings and large findings, which may misrepresent the body of evidence. In addition, critical appraisal via the GRADE criteria is limited in that no statistical detail is available when assessing parameters of inconsistency and imprecision. This is especially true given that heterogenous measures of morbidity are aggregated into larger outcomes. The certainty in evidence may be overestimated as the threshold for rating down a level is less defined. A limitation of the review process can be attributed to the components of screening, data extraction and critical appraisal being conducted by one reviewer.

## Conclusion

There is no conclusive evidence to suggest a differential burden of morbidity between unilateral and bilateral TEP repairs. The findings from the meta-analysis show no difference in the occurrence of seroma, urinary retention, haematoma and length of hospital stay. This meta-analysis has demonstrated that bilateral TEP repair of inguinal hernia is only associated with a 38% increase in operative time. Although unable to be analyzed via quantitative synthesis, recurrences appear to occur more frequently following bilateral repairs. As all included papers are observational studies, data regarding all outcomes is at best very low-quality evidence. This review highlights a need for randomized controlled trials to be conducted in this area to improve the quality of evidence available.

### Other information

This systematic review and meta-analysis was conducted following PRISMA guidelines [50], SWiM guidelines [51] were consulted for syntheses in which a meta-analysis could not be performed.

The protocol for this systematic review and meta-analysis was registered with PROSPERO, registration number: CRD42022309050.

Several amendments have been made to the protocol since its registration. Quantitative synthesis was conducted in R as opposed to REVMAN as this better suited the expertise of the Statistical Consultant. The intended measure of effect was changed from Odds Ratio (OR) to Risk Ratio (RR) for dichotomous outcomes. Ratio of Means (ROM) was added as a measure of effect for continuous outcomes. Vote counting was added as a synthesis method for outcomes in which a meta-analysis could not be performed. No other deviations from the protocol occurred.

### Supplementary Information

Below is the link to the electronic supplementary material.

Supplementary file1 (PDF 367 KB)
