# Supplementary material for: Comparison of bilateral to unilateral total extra-peritoneal (TEP) inguinal hernia repair: a systematic review and meta-analysis
Source: Hernia. 2023 Apr 3;27(5):1047–57. doi: 10.1007/s10029-023-02785-0 (PMC10533595; doi:10.1007/s10029-023-02785-0)
Supplement: Supplementary file 1 — Supplementary file1 (PDF 367 KB) [file 10029_2023_2785_MOESM1_ESM.pdf]

TITLE

Comparison of bilateral to unilateral total extra-peritoneal (TEP) inguinal hernia repair: A systematic review and meta-analysis.

JOURNAL

Hernia.

AUTHORS

1. Thomas Hitman (Corresponding author). University of Auckland, School of Medicine, Auckland, New Zealand. thit441@aucklanduni.ac.nz. ORCID: 0000-0002-9953-4381
2. A S R Bartlett. Laparoscopy Auckland, Epsom, Auckland, University of Auckland, Department of Surgery, and Department of General Surgery, Auckland City Hospital, Grafton, Auckland.
3. Andrew Bowker. Laparoscopy Auckland, Epsom, Auckland, New Zealand.
ORCID: 0000-0002-0260-5156
4. Jessica McLay. University of Auckland, Faculty of Science, Statistics, Auckland, New Zealand.

**Supplementary Table 1.** Outcomes		
**Outcome**		**Definition**
1.	Operative time	The duration from the beginning of the operation to its completion, measured in minutes.
2.	Conversion to open	The event in which the laparoscopic procedure is transitioned to an open method (eg. Lichtenstein) due to a perceived greater likelihood of success.
3.	Intraoperative complications	Surgical complications occurring during the operation, not otherwise specified.
4.	Seroma	The occurrence of a postoperative serous fluid collection in the space left behind the dissected hernia sac, present on clinical examination.
5.	Urinary retention	The event in which a patient requires bladder decompression due to an inability to void following the operation.
6.	Haematoma	The occurrence of a postoperative blood collection in an extravascular space, present on examination as a palpable localized mass and/or visible bruising.
7.	Misc. postoperative complications	Surgical or general complications occurring after the completion of the operation, not otherwise specified.
8.	Recurrence	The occurrence of an inguinal hernia at the site previously repaired.
9.	Post-op pain at 24hr and 7 days	Pain assessed on a given scale, at intervals of 24hrs and 7 days following the operation.
10.	Misc. measures of postoperative pain	Assessments of postoperative pain not otherwise specified.
11.	Length of hospital stay	The period in time from admission to discharge, measured in days.
12.	Time to return to work	The period in time from the operation to the day that work is recommenced, measured in days.
13.	Time to return to ADLs	The period in time from the operation until normal activities of daily living (ADLs) – eg. showering, cooking, self-cares, are met by the patient, measured in days.

**Supplementary Table 2.** Study characteristics and pre-operative patient data.										
							Study arm, no. of patients			
Study (year)	Study location	Study design	Mean age, yr. B/U	Male sex, % B/U	Mean ASA score B/U	Total patients	Bilateral TEP	Unilateral TEP	Major outcome measures	Mean Follow-up duration, (range)
Lau (2003) [1]	Hong Kong	Ret, single centre, Cohort.	66.8	98.1/94.2	NR	206	103	103	Seroma, urinary retention, bruising, wound dehiscence, vas deferens division, recurrence, convalescence period, mean length of stay, operative time.	7 months (0.25-31.2)
Bochkarev (2007) [2]	USA, Nebraska	Pros, single centre, Cohort.	46.8*	100	NR	100	22	78	Seroma, spermatic cord haematoma, urinary retention, hypoxia, postoperative pain, recurrence, time to normal activity, chronic pain at 6 weeks, operative time.	24.5* months (4-46)
Ismail (2010) [3]	India	Ret, single centre, Cohort.	46.3/45.0	99.6/93.3	NR	929	825	104	Seroma, urinary retention, postoperative pain, mean length of stay, recurrence, conversion, operative time.	25.5* months (12-40)
Choi (2011) [4]	Korea, Seoul	Ret, single centre, Cohort.	56.0/47.0	96.4/90.9	NR	879	112	767	Seroma, postoperative pain, recurrence, mean length of stay, operative time.	38 months (3-63)
Gass (2012) [5]	Switzerland	Pros, population-based analysis.	54.6/53.6	96.3/94.6	1.50/1.40	6505	3048	3457	Intraoperative complications, postoperative complications, conversion, operative time, mean length of stay, operative time.	NR
Köckerling (2015) [6]	Germany, Austria, Switzerland	Pros, population-based analysis.	56.1/55.0	94.6/87.5	1.83/1.80	9395	2695	6700	Intraoperative complications: bleeding, injuries. Postoperative complications: bleeding, seroma, impaired wound healing, infection. Operative time, mean length of stay, reoperations.	NR
Tiwary (2020) [7]	India, Varanasi	Pros, single centre, Cohort.	40.5	100	NR	30	2	28	Wound infection, haematoma, testicular discomfort, oedema of cord, inguinodynia, mean length of stay, time to return to work, mean VAS (24hr), operative time.	12 months (12-12)
Kebabci (2021) [8]	Turkey, Izmir	Ret, single centre, Cohort.	48.4/49.4	98.1/92.9	NR	109	53	56	Seroma, haematoma, hydrocele, testicular pain, recurrence, mean length of stay, operative time.	NR
B/U, bilateral/unilateral; NR, not reported; Pros, Prospective; Ret, Retrospective.										
* Mean estimated from the reported median and range in accordance with [9].										

Supplementary Table 3. GRADE evidence and summary of findings table.

CI: confidence interval; **MD:** mean difference; **RR:** risk ratio

Certainty assessment							№ of patients		Effect		Certainty	Importance
№ of studies	Study design	Risk of bias	Inconsistency	Indirectness	Imprecision	Other considerations	bilateral TEP	unilateral TEP	Relative (95% CI)	Absolute (95% CI)		
**Operative time (assessed with: minutes)**												
8	observational studies	not serious	not serious[a]	not serious	not serious	none	6860	11293	-	ROM **1.38 higher** (1.31 higher to 1.45 higher)	⊕⊕◯◯ Low	IMPORTANT
**Conversion to open**												
2	observational studies	not serious	not serious	not serious	very serious[b]	none	34/3873 (0.9%)	33/3561 (0.9%)	**RR 1.08** (0.67 to 1.75)	**1 more per 1,000** (from 3 fewer to 7 more)	⊕◯◯◯ Very low	CRITICAL
**Seroma**												
6	observational studies	not serious	not serious	not serious	serious[c]	none	83/3885 (2.1%)	113/7883 (1.4%)	**RR 1.32** (0.96 to 1.81)	**5 more per 1,000** (from 1 fewer to 12 more)	⊕◯◯◯ Very low	CRITICAL
**Urinary retention**												
3	observational studies	not serious	serious[d]	not serious	very serious[e]	none	48/1025 (4.7%)	11/360 (3.1%)	**RR 1.17** (0.36 to 3.80)	**5 more per 1,000** (from 20 fewer to 86 more)	⊕◯◯◯ Very low	CRITICAL
**Haematoma**												
2	observational studies	not serious	not serious	not serious	very serious[f]	none	5/233 (2.1%)	4/262 (1.5%)	**RR 1.29** (0.36 to 4.67)	**4 more per 1,000** (from 10 fewer to 56 more)	⊕◯◯◯ Very low	CRITICAL
**Recurrence**												
5	observational studies	not serious	serious[g]	not serious	not serious	none	4 of 5 studies saw an increased rate of recurrences following bilateral repairs; 1 study following unilateral repairs.				⊕◯◯◯ Very low	CRITICAL
**Length of hospital stay (assessed with: days)**												
4	observational studies	not serious	not serious[h]	not serious	not serious	none	5958	11027	-	ROM **1.08 higher** (1.00 higher to 1.16 higher)	⊕⊕◯◯ Low	IMPORTANT

**Supplementary Table 4.** Intraoperative outcomes					
	Total patients	Study arm, no. of patients		Outcomes	
		Bilateral TEP	Unilateral TEP	Bilateral TEP	Unilateral TEP
**Mean operative time, mins**					
Lau (2003)	206	103	103	97.00 ± 35.4	64.00 ± 26.10
Bochkarev (2007)	100	22	78	53.90 ± 43.56	38.70 ± 22.20
Ismail (2010)	929	825	104	31.30 ± 5.50	23.70 ± 5.50
Choi (2011)	879	112	767	33.00 ± 12.48	22.39 ± 9.09
Gass (2012)	6505	3048	3457	86.00 ± 36.00	67.00 ± 31.0
Köckerling (2015)	9395	2695	6700	60.30 ± 42.43	44.70 ± 18.35
Tiwary (2020)	30	2	28	120.00 ± 42.43	99.29 ± 25.08
Kebabci (2021)	109	53	56	86.00 ± 37.52	54.50 ± 34.33
**Conversion to open, events (%)**					
Ismail (2010)	929	825	104	2 (0.24%)	0 (0.00%)
Gass (2012)	6505	3048	3457	32 (1.05%)	33 (0.95%)
**Intraoperative complications:**					
**Unspecified, events (%)**					
Gass (2012)	6505	3048	3457	94 (3.08%)	65 (1.88%)
**Injuries, events (%)**					
Lau (2003)	206	103	103	0 (0.00%)	1 (0.97%)
Vas deferens division				0 (0.00%)	1 (0.97%)
Köckerling (2015)	9395	2695	6700	30 (1.11%)	42 (0.63%)
Vascular				8 (0.30%)	16 (0.24%)
Bowel				1 (0.04%)	4 (0.06%)
Bladder				7 (0.26%)	3 (0.04%)
Nerve				0 (0.00%)	1 (0.01%)
**Bleeding, events (%)**					
Köckerling (2015)	9395	2695	6700	16 (0.59%)	53 (0.79%)

Supplementary Table 5. Postoperative outcomes.					
	Total patients	Study arm, no. of patients		Outcomes	
		Bilateral TEP	Unilateral TEP	Bilateral TEP	Unilateral TEP
**Seroma, events (%)**					
Lau (2003)	206	103	103	9 (8.74%)	9 (8.74%)
Bochkarev (2007)	100	22	78	3 (13.64%)	7 (8.97%)
Ismail (2010)	929	825	104	35 (4.24%)	6 (5.77%)
Choi (2011)	879	112	767	13 (11.61%)	55 (7.17%)
Köckerling (2015)	9395	2695	6700	21 (0.78%)	34 (0.51%)
Kebabci (2021)	109	53	56	2 (3.77%)	2 (3.57%)
**Urinary retention, events (%)**					
Lau (2003)	206	103	103	4	3
Bochkarev (2007)	100	22	78	1	0
Ismail (2010)	929	825	104	43	8
**Haematoma, events (%)**					
Lau (2003)	206	103	103	5 (4.85%)	3 (2.91%)
Tiwary (2020)	30	2	28	0 (0.00%)	0 (0.00%)
Kebabci (2021)	109	53	56	0 (0.00%)	1 (1.79%)
**Misc. post-op complications, events (%)**					
Lau (2003)	206	103	103		
Wound dehiscence				1 (0.97%)	0 (0.00%)
Bochkarev (2007)	100	22	78		
Spermatic cord haematoma				1 (4.55%)	4 (5.13%)
Hypoxia				1 (4.55%)	0 (0.00%)
Gass (2012)	6505	3048	3457		
Surgical postoperative complications				96 (3.15%)	78 (2.30%)
General postoperative complications				29 (0.95%)	25 (0.72%)
Köckerling (2015)	9395	2695	6700		
Intestinal lesion				1 (0.04%)	0 (0.00%)
Intestinal obstruction				1 (0.04%)	0 (0.00%)
Impaired wound healing				3 (0.11%)	9 (0.13%)
Bleeding				25 (0.93%)	77 (1.15%)
Wound infection				1 (0.04%)	3 (0.04%)
Reoperations				48 (1.78%)	55 (0.82%)

Tiwary (2020)		30	2	28		
Oedema of cord					0 (0.00%)	2 (7.14%)
Wound infection					0 (0.00%)	0 (0.00%)
Testicular pain					0 (0.00%)	0 (0.00%)
Kebabci (2021)		109	53	56		
Hydrocele					0	1 (1.79%)
Testicular pain					11 (20.80%)	13 (23.20%)
**Recurrence, events (%)**						
	Mean follow-up, months (range)					
Lau (2003)	7 (0.25-31.2)	206	103	103	1 (0.97%)	0 (0.00%)
Bochkarev (2007)	24.5 (4-46)	100	22	78	2 (9.09%)	0 (0.00%)
Ismail (2010)	25.5 (12-40)	929	825	104	2 (0.24%)	0 (0.00%)
Choi (2011)	38 (3-63)	879	112	767	1 (0.89%)	0 (0.00%)
Tiwary (2020)	12 (12-12)	30	2	28	0 (0.00%)	0 (0.00%)
Kebabci (2021)	NR	109	53	56	3 (5.66%)	2 (3.57%)
**Post-op pain**						
Ismail (2010)		929	825	104		
Mean post-op pain† at 24hrs					2.21 ± 0.4	2.19 ± 0.4
Mean post-op pain† at 1 week					1.20 ± 0.4	1.24 ± 0.5
Tiwary (2020)		30	2	28		
Mean VAS score in first 24hr					1.50 ± 0.70	1.36 ± 0.62
Bochkarev (2007)		100	22	78		
Post-op pain >2 weeks, events (%)					2 (9.09%)	2 (2.56%)
Chronic pain at 6 weeks, events (%)					0 (0.00%)	0 (0.00%)
Choi (2011)		879	112	767		
>2 doses of analgesics, events (%)					12 (10.71%)	64 (8.34%)
Tiwary (2020)		30	2	28		
Inguinodynia, events (%)					0 (0.00%)	0 (0.00%)
**Mean length of hospital stay, days**						
Lau (2003)		206	103	103	1.70 ± 1.10	1.50 ± 1.60
Ismail (2010)		929	825	104	1.07 ± 0.30	1.09 ± 0.30
Choi (2011)		879	112	767	0.89 ± 0.43	0.90 ± 0.47
Gass (2012)		6505	3048	3457	2.50 ± 7.00	2.30 ± 6.40

Köckerling (2015)	9395	2695	6700	1.80 ± 0.95	1.60 ± 1.05
Tiwary (2020)	30	2	28	1.00	1.00
Kebabci (2021)	109	53	56	1.00	1.00
**Mean time to return to work, days (range)**					
Bochkarev (2007)	100	22	78	8.4* (6-14)	6.2* (5-8)
Tiwary (2020)	30	2	28	7.50 ± 0.70	4.86 ± 0.83
**Mean time to return to ADLs, days (range)**					
Lau (2003)	206	103	103	3.5 ± 1.8	3.8 ± 2.5
**Hospital stay ≥ 23h, events (%)**					
Bochkarev (2007)	100	22	78	2 (9.09%)	0 (0.00%)
* Standard Deviation (SD) not available.					
† Graded against an internal system for assessing post-operative pain.					
NR, not reported; VAS, Visual Analogue Score for pain.					

REFERENCES:

1. Lau, H., Patil, N.G., Yuen, W.K. (2003). A comparative outcome analysis of bilateral versus unilateral endoscopic extraperitoneal inguinal hernioplastics. *Journal of Laparoendoscopic & Advanced Surgical Techniques, 13*(3), 153-7.
   DOI: 10.1089/109264203766207663.

2. Bochkarev, V., Ringley, C., Vitamvas, M., Oleynikov, D. (2007). Bilateral laparoscopic inguinal hernia repair in patients with occult contralateral inguinal defects. *Surgical Endoscopy, 21*(5), 734-6.
   DOI: 10.1007/s00464-007-9196-x.

3. Ismail, M., Nair, S., Garg, P. (2010). Is prophylactic laparoscopic total extraperitoneal inguinal hernia repair on the contralateral side justified in less developed regions? A comparative study of bilateral to unilateral repair. *Journal of Laparoendoscopic & Advanced Surgical Techniques, 20*(6), 533-6.
   DOI: 10.1089/lap.2009.0453.

4. Choi, Y.Y, Hur, K.Y. (2011). Simultaneous laparoscopic totally extraperitoneal repair of bilateral inguinal hernia: review of 1 surgeon experiences. *Surgical Laparoscopy Endoscopy & Percutaneous Techniques, 21*(4), 264-6.
   DOI: 10.1097/SLE.0b013e31822177fd.
5. Gass, M., Rosella, L., Banz, V., Candinas, D., Güller, U. (2012). Bilateral total extraperitoneal inguinal hernia repair (TEP) has outcomes similar to those for unilateral TEP: population-based analysis of prospective data of 6,505 patients. *Surgical Endoscopy, 26*(5), 1364-8.
   DOI: 10.1007/s00464-011-2040-3.

6. Köckerling, F., Schug-Pass, C., Adolf, D., Keller, T., Kuthe, A. (2015). Bilateral and Unilateral Total Extraperitoneal Inguinal Hernia Repair (TEP) have Equivalent Early Outcomes: Analysis of 9395 Cases. *World Journal of Surgery, 39*(8), 1887-94. DOI: 10.1007/s00268-015-3055-z.

7. Tiwary, S.K., Kumar, S., More, R., Shankar, V., Kumar, S., Dwivedi, AND. (2020). A study of contralateral occult inguinal hernia in adult male patients undergoing total extraperitoneal herniorraphy. *Journal of Family Medicine and Primary Care, 9*(6), 2975-2979.
   DOI: 10.4103/jfmpc.jfmpc_207_20.

8. Kebabci, E., Ozturk, S., Unver, M. (2021). Outcomes of Endoscopic Totally Extraperitoneal (TEP) repair of clinically occult inguinal hernia diagnosed with ultrasonography. *Polski Przeglad Chirurgiczny, 93*(4), 11-14.
   DOI: 10.5604/01.3001.0014.8695.

9. Hozo, S.P. Djulbegovic, B., Hozo, I. (2005). Estimating the Mean and Variance from the Median, Range, and the Size of a Sample. *BMC Medical Research Methodology, 5*(13).
   DOI: 10.1186/1471-2288-5-13.
